# Factors affecting medical students’ satisfaction with online learning: a regression analysis of a survey

**DOI:** 10.1186/s12909-023-04995-7

**Published:** 2024-01-03

**Authors:** Özlem Serpil Çakmakkaya, Elif Güzel Meydanlı, Ali Metin Kafadar, Mehmet Selman Demirci, Öner Süzer, Muhlis Cem Ar, Muhittin Onur Yaman, Kaan Can Demirbaş, Mustafa Sait Gönen

**Affiliations:** 1 Cerrahpaşa Medical Faculty, Department of Medical Education, İstanbul University-Cerrahpaşa, İstanbul, Fatih 34098 Türkiye; 2 Cerrahpaşa Medical Faculty, Department of Histology and Embryology, İstanbul University-Cerrahpaşa, İstanbul, Türkiye; 3 Cerrahpaşa Medical Faculty, Department of Neurosurgery, İstanbul University-Cerrahpaşa, İstanbul, Türkiye; 4 Cerrahpaşa Medical Faculty, Department of Anatomy, İstanbul University-Cerrahpaşa, İstanbul, Türkiye; 5 Cerrahpaşa Medical Faculty, Department of Medical Pharmacology, İstanbul University-Cerrahpaşa, İstanbul, Türkiye; 6 Cerrahpaşa Medical Faculty, Department of Internal Medicine, Division of Haematology, İstanbul University-Cerrahpaşa, İstanbul, Türkiye; 7 Vocational School of Health Services, Department of Medical Services and Techniques, İstanbul University-Cerrahpaşa, İstanbul, Türkiye; 8 Cerrahpaşa Medical Faculty, İstanbul University-Cerrahpaşa, İstanbul, Türkiye; 9 Cerrahpaşa Medical Faculty, Department of Internal Medicine, Division of Endocrinology, İstanbul University-Cerrahpaşa, İstanbul, Türkiye

**Keywords:** Medical education, Online learning, Students’ satisfaction, Linear regression analysis

## Abstract

**Background:**

Medical education requires the implementation of different teaching methods and strategies for future doctors to achieve broad learning objectives. This wide range of methods and strategies includes the use of Information Technologies. For a long time, there was a call for a change in medical education for blending new teaching approaches to lessen medical students’ class time. The COVID-19 pandemic then sped up the transition to the new way of medical education and classroom lectures were quickly moved to a virtual environment. We expect that these changes will continue, and online learning will be one of the main teaching strategies in medical education. Therefore, educational experiences during the COVID-19 pandemic will improve our understanding of online learning and will help to develop blended medical school curricula in the future. For this reason, we aimed to determine students’ overall satisfaction with their online learning experience and to define the main factors affecting students’ satisfaction with their online learning program at Cerrahpaşa Medical Faculty.

**Methods:**

A cross-sectional survey study was conducted to determine medical students’ overall satisfaction with online learning methods and to identify factors associated with positive and negative satisfaction levels. A questionnaire, consisting of 24 questions to collect demographic characteristics, factors associated with online education experience and overall satisfaction levels was developed and distributed to 1600 medical students. Multivariable linear regression analysis was used to determine the factors associated with positive and negative satisfaction levels.

**Results:**

Regression analysis showed that being familiar with online teaching techniques (*β* = 0.19, 95% CI [0.07, 0.30], faculty members’ higher online teaching skill levels (*β* = 0.42, 95% CI [0.32, 0.51], interactive online teaching approaches (*β* = 0.54, 95% CI [0.41, 0.67], having a personal workspace (*β* = 0.43, 95% CI [0.19, 0.67], and a self-reported longer attention span (*β* = 0.75, 95% CI [0.57, 0.92] were associated with higher overall satisfaction with online learning. The occurrence of technical problems (*β* = **-**0.19, 95% CI [**-**0.26, **-**0.12] was associated with lower overall satisfaction.

**Conclusions:**

Higher online teaching skills of faculty members, use of interactive approaches, students’ familiarity with online teaching techniques, provision of a personal workspace, and self-reported longer attention spans positively contributed to higher levels of student satisfaction with online learning. Considering the increasing significance of online educational methods, our study identified key components that affect students’ level of satisfaction. This information might contribute to the development of online educational programs in the future.

## Background

Graduating successful clinicians from medical schools is a very complex process. This complexity demands the implementation of different teaching methods and strategies for medical students to achieve broad learning objectives. In 2010, the Lancet Commission underscored the importance of the use of all learning channels to their full potential to facilitate transformative changes of health professionals’ education. Those learning channels explicitly included the use of Information Technologies (IT) [1, 2]. Online learning was seen as a revolution although some barriers were recognized such as unequal distribution of digital resources. Additionally, institutional transformation to implement this new teaching method was stated as an inevitable element of online learning. That transformation does not only include software or hardware but also the need for humanware [1]. The Lancet Commission recommendations were embedded in a two decades long call for change in medical education. Online learning methods had already gained in popularity, because of relatively low costs, high flexibility, reduced dependence on geographical boundaries, and programs started to rapidly increase in number [3].

When the COVID-19 pandemic hit after two decades of increasing calls, it catalyzed the recommended and expected change [4]. Infection control measures during the pandemic forced medical schools moving to Emergency Remote Teaching [5–7].

In Türkiye the first COVID-19 case was seen on the 16th of March 2020 just after the declaration of the World Health Organization’s COVID-19 outbreak as global pandemic. Educational institutions were closed due to lockdowns. Therefore, development and implementation of Emergency Remote Teaching, which means a temporary shift of instructional delivery to an alternate delivery mode due to crisis circumstances, became mandatory [8].

According to the Association for Evaluation and Accreditation of Medical Education Programs (TEPDAD) report in 2021, at the beginning of the pandemic Emergency Remote Teaching was provided mainly as synchronous, asynchronous, or mixed online didactic lectures at medical schools [9]. Although student-centred education activities such as small group teaching or Problem Based Learning activities could not be carried out to a great extent at the beginning of the pandemic, they were gradually being re-implemented synchronously. Medical schools tried to continue education with the tools available while IT infrastructure improvements were implemented over time. Nevertheless, deficiencies in clinical training and hands-on clinical skills training were the main concerns during the pandemic [9].

By the beginning of the COVID-19 pandemic, face-to-face education suddenly had to turn to Emergency Remote Teaching at Cerrahpaşa Medical Faculty just like many medical schools in Türkiye and around the world [10]. We started to use a Learning Management System (CANVAS Learning Management System, Instructure, Salt Lake City, UT, USA) for online education and adapted the system to our educational platform. The system included an overall course page, content page that included previously recorded lectures, slideshows and learning materials, an announcement page, and an assignment and exam page. Lectures were given as synchronous live streaming, and additionally, they were recorded to provide asynchronous learning options. Students were able to access the presentations and preparation materials beforehand. All students were assigned a personal ID for access to the system.

This method was fairly new for our faculty members and students, and it caused struggling during the mandatory transition. Therefore, we implemented some emergency solutions for facilitating their adaptation. Firstly, we established an online learning support team (Distance Education Committee). The team worked with the Undergraduate Medical Education Program Evaluation and Development Commission continuously on the organization of online learning. Additionally, this committee acted as a bridge between Medical School and the University IT Department on technical issues. Secondly, we provided lectures to faculty members on the use of the Learning Management System, pedagogic formation for the digital environment, and online evaluation and assessment methods.

This emergency implementation required the timely evaluation of this new method to enable and ensure the development and growth of the system without delay. For this program evaluation, we focused on factors that have been shown to affect students’ satisfaction, such as instructional attributes, computer self-efficacy, ease of use, and accessibility [11]. In addition, we measured the overall satisfaction score as a reflection of the students’ responses to their learning experience. Satisfaction in the context of higher education can be described as an attitude resulting from students’ educational experience, services and facilities provided by the institution [12]. As an indirect feedback factor it gives the institution an opportunity to improve and modify their methods according to the students’ needs, and as a direct factor it influences the involvement, trust, and loyalty of students toward their curriculum and facilities [13]. In the literature, there are several studies connecting student satisfaction with favourable outcomes both in medical and non-medical training programs [14–16]. In these studies, student satisfaction level was positively correlated with persistence in learning, motivation, and significantly higher levels of knowledge, involvement, and trust [13, 17, 18]. In addition, learner satisfaction or reaction to the program is the first level in Kirkpatrick’s four-level program evaluation model and is a suggested measure in formal guidelines on program evaluation models [19, 20]. Therefore, student satisfaction is an important endpoint in formal program evaluation. Understanding modifiable factors that influence the learning experience, measured as students’ overall satisfaction, is essential to improve and optimize online learning and to deliver exactly to the students’ needs [19, 21, 22].

Therefore, we designed this study for assessing students’ overall satisfaction with Emergency Remote Teaching and for defining the main factors that contribute to students’ satisfaction in the online environment at Cerrahpaşa Medical Faculty. Additionally, we aimed to understand what kind of challenges students faced during online learning.

Following, we will use the term ‘online learning’ instead of ‘Emergency Remote Teaching’ throughout the manuscript since we used this term in the questionnaire; unless it is specified as Emergency Remote Teaching in a cited reference in which case the original term of the cited reference will be used.

## Methods

A cross-sectional survey study was conducted to determine medical students’ overall satisfaction with online learning methods. Factors associated with positive and negative satisfaction levels were identified using multivariable linear regression analysis. The study consisted of three phases: (1) Planning and designing the research study including questionnaire development and validation; (2) Data collection; and (3) Data analysis and interpretation.

The study was designed and conducted in accordance with the principles of the Declaration of Helsinki. Ethics Committee of the Cerrahpaşa Medical Faculty approved the study (Number: 13549728-619-169921). At the beginning of the questionnaire, informed consent was obtained after the students were informed about the purpose and length of the questionnaire. All students were over the age of 18.

### Target population

Undergraduate medical education takes six years in our country. According to the medical school curriculum, the first three years are considered pre-clinical, and the 4th and 5th years are clinical. The last year is an internship period. Cerrahpaşa Medical Faculty has two different programs based on primary teaching language, a Turkish and an English program. Students of both programs and from the first five years were invited to answer the questionnaire. Since sixth year’s students worked clinically during the pandemic they did not receive online education. Therefore, the target population was all Cerrahpaşa Medical Faculty students studying online in the 2020 academic year spring semester.

### Development of the questionnaire

We conducted an extensive literature review before determining the questions. Additionally, we examined guidelines and checklists to improve the quality of the questionnaire [23, 24]. During the development and implementation of the survey, we specifically considered the Checklist for Reporting Results of Internet E-Surveys (CHERRIES) [24].

The faculty members of the Department of Medical Education determined the items of the questionnaire. Additionally, we discussed the questions during a focus meeting and via e-mail exchanges with the members of the Cerrahpaşa Medical Faculty’s Survey Evaluation Board and the Distance Education Committee. Cerrahpaşa Medical Faculty’s Survey Evaluation Board has four faculty members (from Medical Education, Anatomy, Neurosurgery, and Histology Departments) who create questionnaires and organize the administration of surveys since 2012. The Distance Education Committee was established and started to work at the beginning of pandemic. There are five faculty members in this commission from different departments. A standard expert-panel process with seven faculty members from different educational committees confirmed the face validity of all items of the questionnaire. Experts agreed that the items of the test are appropriate, clearly expressed, and relevant to online education. All of them confirmed that the clarity of wording, layout, and style of the questionnaire are adequate.

The questionnaire consisted of three different parts with 24 questions. The first part included demographic items (five questions). The second part consisted of eleven statements regarding teaching methods, technical opportunities, and attitudes toward online education, which were rated on a 5-point Likert scale. The third part of the questionnaire consisted of open-ended questions, which aimed to determine the strengths, weaknesses, and developmental aspects of online learning (seven questions). Additionally, we asked for the overall satisfaction score as the primary outcome of the study, which was measured on a 10-point scale [1–10] (Question: How would you rate your overall satisfaction level on a scale from 1 to 10 with the online education at Cerrahpaşa Medical Faculty? (1: I am not satisfied at all; 10: I am very satisfied)).

Each question was placed on one screen. All items had a non-response option and selection of one response was enforced. Participants were able to review and change their answers through a back button.

The LimeSurvey survey tool (LimeSurvey GmbH, Hamburg, Germany) was used for creating the online survey and collecting data.

### Pre-testing

We tested the electronic questionnaire before its submission by sending the survey link to student representatives from each class of two programs (10 students). They completed the pilot test and checked the completeness.

### Data collection

After the completion of the 2020 academic year spring semester, we announced the survey by e-mail and Short Message Service (SMS) and provided a link; 1600 medical students received the questionnaire via SMS and e-mail. The link was active during a two-week period. Completing the questionnaire was voluntary and no incentives were offered. IP addresses of the respondents’ electronic device were used to identify potential duplicate entries from the same user. Once the questionnaire was completed, the system did not allow the user to submit additional entries. However, if the questionnaire was not completed, additional entries were allowed. In these cases, entries from the same IP addresses were eliminated. The most recent questionnaires were kept for analysis.

### Statistical analysis

We first investigated the bivariable unadjusted association between the primary outcome (overall satisfaction) and 14 items from the questionnaire, using univariable regression. To identify possible items that predicted students’ satisfaction scores, we developed a linear regression model with students’ overall satisfaction scores as the dependent variable and 14 different items from the questionnaire as possible predictors. We used a backwards stepwise regression approach to select the most significant predictors at each iteration for simplifying our model. Variables with a p-value threshold above 0.1 were excluded from the model. Statistical significance was assumed below a p-value threshold of 0.05.

For strategic planning purposes of our future online education programs, we summarized open-ended questions with SWOT (Strengths, Weaknesses, Opportunities, and Threats) analysis. For this analysis, we asked four open-ended questions.

1. What are the most important strengths of online learning you experienced this semester?
2. What are the negative/weak sides of online learning you experienced this semester?
3. Which areas do need improvement (or should be avoided)?
4. Please let us know if you have any other thoughts.

We followed an inductive process. Survey Evaluation Board members read all answers to open-ended questions and then coded the data. They identified the patterns and themes within the responses. After all, we held a consensus meeting to ensure that the themes identified were relevant to the SWOT analysis.

The sample size of this study was given by the number of enrolled students at the time of the study and the number of survey responses.

We are reporting the results of our survey in accordance with the Checklist for Reporting Results of Internet E-Surveys (CHERRIES) [24].

We used Stata 14.2 (Stata Corp., USA) for all statistical analyses.

## Results

### Response rates

The number of respondents who entered the survey was 1117 out of 1600 medical students. Sixty-seven non-completed questionnaires were excluded from the analysis because of double entries from the same IP addresses. Therefore, the survey had 1050 individual respondents. One hundred thirty respondents stopped to answer the questionnaire at later questions. The number of fully completed surveys was 920. Fully completed and early-terminated questionnaires were included in the analysis.

The response rate of the questionnaire was 66% (1050/1600). The completion rate was 88% (920/1050). Table 1 shows respondent characteristics and overall satisfaction score.

**Table 1:** Students’ characteristics and overall satisfaction score

Students’ Characteristics	Total Population (n = 1050)
Age	21.4 ± 3.5
Gender (Female/Male)	458/564
Program (English/Turkish Program)	199/850
Phase (Preclinical/Clinical)	541/507
Students who have their own internet access	972 (94%)
Students who have their personal workspace	806 (78%)
Mostly used devices for e-learning	
♣ Laptop	498 (47%)
♣ Cell Phone	435 (42%)
♣ Tablet	64 (6%)
♣ Desktop	32 (3%)
♣ Others	19 (2%)
The overall satisfaction score	6.42 ± 2.16
Results are given in number (%) or mean ± standard deviation	

### Results of the Likert scale statements

**Fig. 1:**
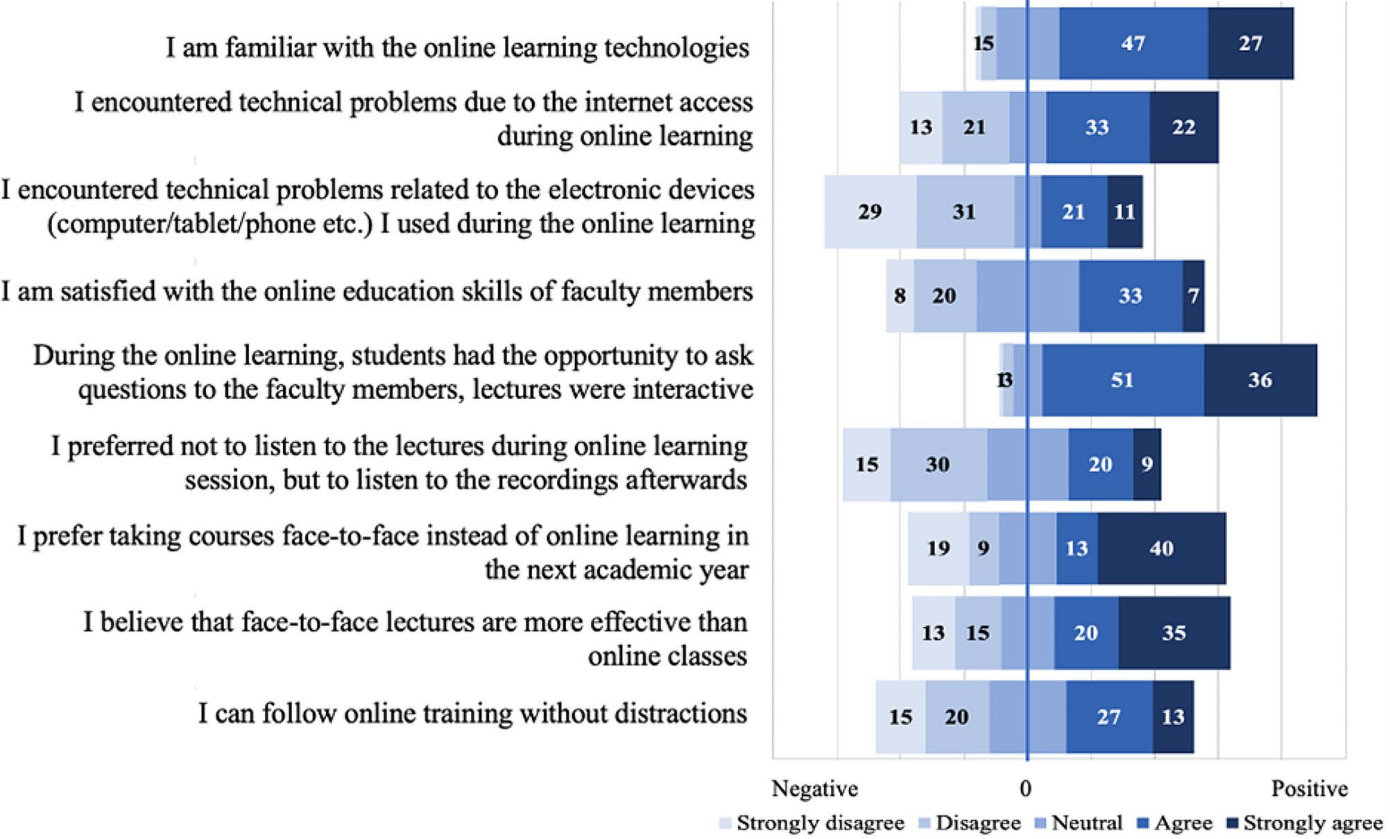
Results of likert-type of questions (numbers indicate percentages)

Likert-type questions were categorized as individual characteristics, technical opportunities, teaching methods, and attitudes toward online education. Percentages of answers per Likert category for the items measured on a 5-point Likert scale are displayed in Fig. 1.

**Fig. 2:**
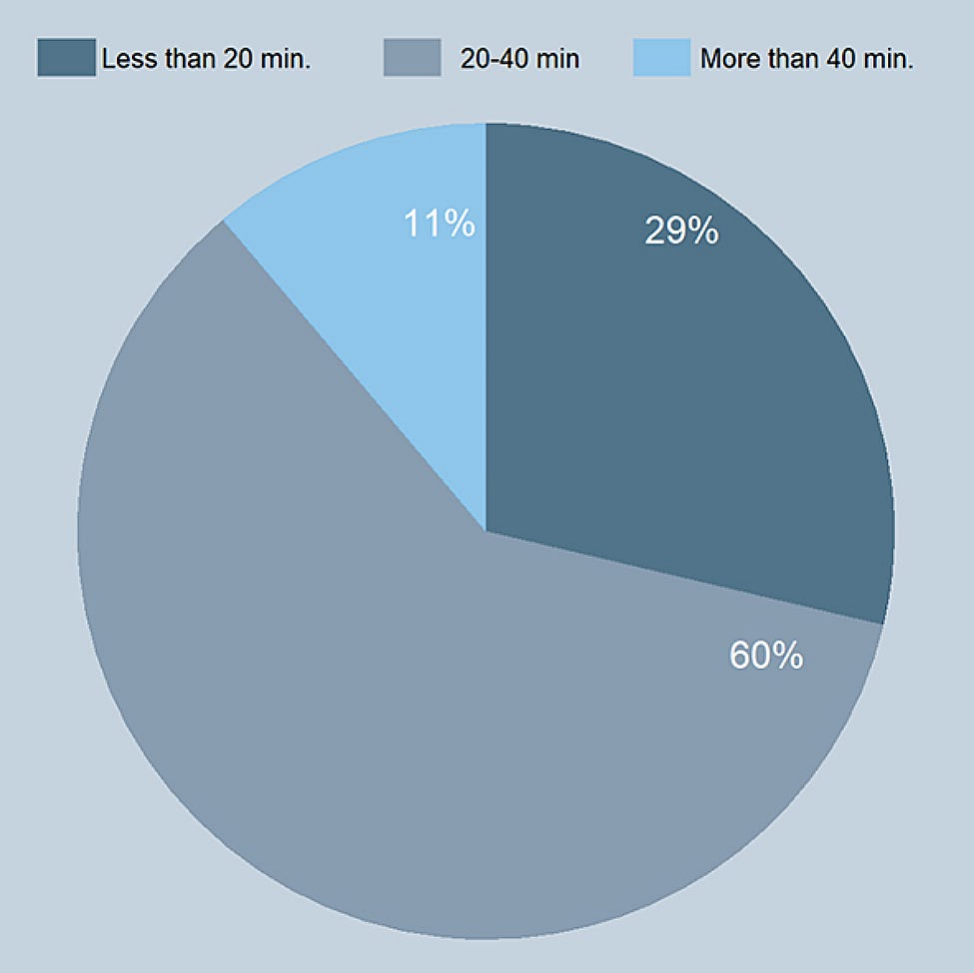
Students’ optimum focusing time during the online lectures

In addition to the statement “I can follow online training without distraction”, we asked for the students’ optimum focusing time during the online lectures. Most students (60%) reported it as 20–40 min (Fig. 2).

### Linear regression analysis

In unadjusted bivariable analysis, 12 of the 14 potentially predicting survey items were significantly associated with students’ overall satisfaction. In the adjusted linear regression model, seven of the 14 entered items were significantly associated with students’ overall satisfaction. In the final adjusted stepwise regression model, eight items were kept in the model (p-value threshold of 0.1) and seven of those items (listed below) were significantly associated with students’ overall satisfaction (p-value threshold of 0.05). The final regression model resulted in an adjusted r-squared value of 0.60, indicating that the model explains 60% of the total variability of the observed data.

Positive association with students’ overall satisfaction

- Being familiar with online teaching techniques and reporting good IT literacy.
- Having their own personal workspace.
- Satisfaction with faculty members’ online teaching skills.
- Interactive teaching methods used by faculty members.
- Self-reported longer attention span during online learning.

Negative association with students’ overall satisfaction

- Encountering technical problems.
- Believe that lecture hall lectures are more effective.

The significantly associated items did not differ between the plain regression model and the stepwise regression. Table 2 presents the results of the bivariable analysis, plain linear regression model, and linear regression analysis with backwards predictor selection.

**Table 2:** Results of the linear regression analysis (Bivariable analysis, plain linear regression model, and linear regression analysis with backwards predictor selection)

	Bivariable Analysis			Plain Regression Model			Stepwise Regression		
Variables	Unadjusted Coefficient	(95% CI)	p	Adjusted Coefficient	(95% CI)	p	Adjusted Coefficient	(95% CI)	p
Age (years)	0.09	0.05 to 0.13	< 0.001	0.03	-0.001 to 0.05	0.054	0.02	-0.002 to 0.05	0.072
Gender (Reference: Male)	-0.07	-0.36 to 0.21	0.607	0.05	-0.13 to 0.24	0.566			
Program (Reference: Turkish Program)	0.35	-0.01 to 0.71	0.058	0.17	-0.06 to 0.41	0.154			
Phase (Reference: Preclinical Phase)	0.56	0.28 to 0.84	< 0.001	-0.07	-0.27 to 0.13	0.467			
Easier access to internet connection	1.71	1.14 to 2.28	< 0.001	0.24	-0.15 to 0.64	0.237			
Having own personal workspace	1.85	1.53 to 2.16	< 0.001	0.40	0.15 to 0.64	0.001	0.43	0.19 to 0.67	< 0.001
Experiencing connection problems during the lectures	0.95	0.81 to 1.10	< 0.001	-0.02	-0.11 to 0.06	0.555			
Experiencing technical problem with electronic devices	-0.61	-0.70 to -0.51	< 0.001	-0.17	-0.25 to -0.09	< 0.001	-0.19	-0.26 to -0.12	< 0.001
Being familiar with online teaching techniques and reporting good computer skills (IT literacy)	0.95	0.81 to 1.10	< 0.001	0.18	0.06 to 0.30	0.003	0.19	0.07 to 0.30	0.001
Satisfaction with faculties’ online teaching skills	1.02	0.90 to 1.13	< 0.001	0.40	0.30 to 0.50	< 0.001	0.42	0.32 to 0.51	< 0.001
Interactive teaching methods used by faculties	1.34	1.19 to 1.50	< 0.001	0.54	0.41 to 0.67	< 0.001	0.54	0.41 to 0.67	< 0.001
Preferring asynchronous lectures instead of live lectures	-0.27	-0.38 to -0.15	< 0.001	-0.01	-0.09 to 0.07	0.782			
Believe that face-to-face lectures are more effective	-0.81	-0.89 to -0.73	< 0.001	-0.43	-0.50 to -0.36	< 0.001	-0.43	-0.50 to -0.36	< 0.001
Reporting longer attention span during online learning	2.01	1.81 to 2.20	< 0.001	0.75	0.57 to 0.93	< 0.001	0.75	0.57 to 0.92	< 0.001

### SWOT analysis for program evaluation

We performed a SWOT analysis for strategic planning of future online education programs, using the qualitative data from open-ended questions of the survey (Table 3).

**Table 3:** SWOT analysis evaluation table

SWOT ANALYSIS	
**Strength**	**Weakness**
• Thanks to the recordings, lectures can be listened to again • More efficient use of time (reduced time spent for commuting) • Online learning is safer because it reduces the risk of infection	• Problems related to online learning technical infrastructure • Inadequate or inefficient remote hands-on clinical skills training, clinical training, and lab practices
**Opportunities**	**Threats**
• Implementing online learning into system for lessening medical students’ class time after the pandemic	• Online learning cannot help students for reaching their preclinical and clinical skills and attitude goals

### Example sentences

#### Strength

- It is very useful to record the lectures, the faculty members answered all our questions and the questions asked were recorded. Most importantly, it is necessary for our health.
- During the pandemic, I think it is beneficial to conduct lecture courses remotely. Students’ health is taken care of. No problem hearing or seeing the screen, no distractions from other students around.
- No transportation problem and opportunity to easily ask questions during lectures.
- I saved the time I lost on the way from home to school and was less tired. I was able to devote more time to myself.

### Weakness

- Sometimes there are technical problems in the system and it is difficult to attend the class.
- Since the instructors are new to online teaching, there was a bit of getting used to it at first, but I believe it will be better later.
- The negative effect on the master-apprentice relationship, which is the most basic feature of medical education, and our inability to communicate with our faculty members face to face.
- At that time, you could feel like you are the only person listening to the lecture, this is both a positive and a negative situation.

### Opportunities

- I think it would be for our benefit to integrate the online education into the system not only during the pandemic but also in the normal period.

### Threats

- There is no bedside teaching because of pandemic, we try to be a doctor without seeing patients.
- We cannot become doctors with online education.

## Discussion

In our large survey, we investigated different potentially predictive factors of students’ satisfaction with online learning: Student satisfaction scores have been formally recognized as highly relevant for program evaluation, as they are associated with persistence in learning motivation and significantly higher levels of knowledge, involvement and trust [13, 17, 18]. Our study showed that several, often modifiable conditions and factors predict students’ satisfaction with their online learning experience. Being familiar with online teaching techniques (IT literacy), faculty members’ higher online teaching skill levels, using interactive online teaching approaches, and access to a personal workspace increased overall satisfaction with online learning. The occurrence of technical problems and a self-reported shorter attention span decreased overall satisfaction.

Unsurprisingly, students with lower self-rated IT literacy had lower overall satisfaction scores. In line with this result, the literature has shown the direct effect of students’ computer or internet self-efficacy on course satisfaction [25–27]. A systematic review showed that students' previous online learning experience and being familiar with the system increased their satisfaction level [28]. Similarly, another study found that previous positive experience with the learning platform and students’ level of digital competency has a positive effect on educational satisfaction [29]. Therefore, providing training and support for students in IT literacy might help students to be better prepared for the challenges of digital learning platforms. Computer literacy is not only necessary for facilitating online learning but also it is an integral part of preparing tomorrow’s doctors to be sufficiently competent to use the various informatics resources effectively and efficiently for the best practice of medicine [30]. For this reason, educational programs or courses to teach students how to use the internet and digital technologies had been implemented at some medical schools already long time before the pandemic [31–33].

Our results also showed that the faculty members’ online teaching skills and the use of teaching methods prioritizing interactive strategies increased students’ satisfaction. At the beginning of the pandemic, faculty members were in a hurry to transition to convey the content in a didactic manner; they used didactic live-streamed or recorded lectures. As time passed, we realized the need for more efforts to increase students’ interest and focus. Therefore, faculty members started to use the opportunities of online teaching environments, such as polls and breakout rooms for small group discussions to create an active and student-centred learning environment.

Before the pandemic, the lack of IT-related skills was found to be one of the barriers met by educators when engaging with the development and implementation of online learning [30, 34]. During Emergency Remote Teaching, many teachers realized that teaching online required a reassessment of their teaching strategies and different educational approaches [35]. A meta-synthesis reported that the use of technology without imposing any contemporary pedagogical elements will have little benefit [36]. Therefore, medical schools should ensure that faculty members were provided training and support in combination with creative skills and information navigation before the implementation of an online education program [34, 37]. As an advantage of gaining online teaching skills, learning new educational technologies might be a catalyst for faculty to reflect on and evaluate their current teaching practices. By learning how to teach online, even face-to-face teaching styles can change, and instruction tends to become more student-centred [38]. In line with this, Prober and Heath suggested to change the way we educate doctors and to make better use of our students’ time [39]. They proposed that medical education could be improved without increasing the time to complete a medical degree. In this new approach to medical education, from lecturing to interaction, active learning models gained importance, such as flipped-classrooms and team-based, problem-based, or case-based exercises. Blending these models with the use of online instructions and video lectures to communicate factual material frees up students’ class time.

Even before the pandemic era, attention span during lectures was an important topic of interest [40]. Expert opinions suggested that students’ attention during lectures tends to decrease after approximately 10–15 min, although the evidence is not strong [41, 42]. A survey study from 1978, which assessed medical students’ concentration during lectures showed that their attention rose sharply to reach a maximum after 10–15 min, and fell steadily thereafter, even though the decline was slow [43]. Based on these results, the authors suggested that the optimum length of a lecture might be 30 min instead of 60 min. After so many decades, we now discuss the attention span in the context of online education. According to a recent cross-sectional survey during the pandemic, first year medical undergraduate students stated that their attention span was higher in traditional face-to-face sessions than in online sessions [44]. Another study conducted in medical and dentistry schools showed limited attention span during online lectures [45]. Data regarding maximum attention span, factors affecting attention and inducing distraction among tertiary educational institutions are scant especially in the era of online learning. As sustainability of attention and distraction is a complicated concept that interacts with different external and internal factors, it is not easy to objectively assess and validate the findings on attention span [46]. Further studies and clearer definitions are needed specifically on the topic of attention during online learning. Our students mostly rated their focus time during the online lectures as 20–40 min. For future curricula, we therefore decided to limit the online lectures to 20 min and to use microteaching methods, which have been shown to be more effective compared to traditional online learning modules in undergraduate medical education [47].

Our study identified other modifiable items that were associated with low satisfaction levels with online learning. A well-recognized problem during the pandemic was technical issues with electronic devices and internet connection. These problems were significantly associated with decreased overall satisfaction scores [48]. Moreover, the lack of personal space to study (22%) negatively affected our students’ satisfaction, as the space is an integral factor in minimizing distractions. These results corroborate the decades-old concern that the unequal distribution of digital and environmental resources are barriers to effective online learning as shown similarly in other studies [48–51]. Having a conductive home environment and learning space were reported as important enablers to help students to focus [49]. According to a survey from the UK that included 2721 medical students, family distractions (26.76%), and lack of space (11.03%) were relevant barriers to effective online learning [48]. To address the disparity of resources, and to support our students on technical and workplace issues, we provided computers or internet quota as needed. Additionally, we created some quiet spaces within the existing library to be used for on-campus online learning.

Although many of our students reported some advantages of online teaching such as effective and flexible use of time, infection prevention and opportunity to re-watch the lecture videos after live lectures, most of them stated that face-to-face learning is more effective, and they prefer face-to-face learning to online learning. On the one hand, we understand that online learning cannot replace face-to-face learning, but on the other hand, we do not want to disregard the advantages of online learning in terms of better use of our students’ time. For that reason, we implemented online learning modules to the medical basic skills training for second-year students after we returned to face-to-face teaching in 2022. These modules deliver theoretical knowledge, introduce checklists and give our students more time for hands-on practice when they come to in-person lab sessions. In addition, some of the selective classes also moved to the online platform. Online learning methods have become part of our teaching repertoire now.

A limitation of our study is that the timing of the survey fell into the early months of the pandemic and reflects the experience with online learning when we were rushed to provide continuity of our educational program. Therefore, the results might not be generalizable to post-pandemic well-organized and well-structured online learning activities. However, we believe this experience will improve our understanding of online education and will help to develop medical school curricula in the future. One of the positive gains of the pandemic for us was the establishment of a good Learning Management System and the development of organizational plans not only for future blended learning but also for possible emergencies such as new pandemics or natural disasters.

## Conclusions

When the pandemic increased the speed of the transition from traditional to online learning, we realized that the previous barriers to online learning were not impossible to overcome. In the post-pandemic era, well-planned and developed blended learning programs will increase the students’ satisfaction and will help us to use students’ time more effectively. Our study suggests that using interactive teaching methods, pedagogically improved online teaching skills, adjusting the duration of lectures according to students’ attention span, and addressing the disparity of resources by supporting students on technical and workplace issues might positively affect the students’ satisfaction with our online learning program.

## Data Availability

The datasets and survey questionnaire generated and/or analysed during the current study are not publicly available due to institutional rules of Cerrahpaşa Medical Faculty but are available from the corresponding author on reasonable request.

## Abbreviations

IT: Information Technologies
TEPDAD: Tıp Eğitimi Programlarını Değerlendirme ve Akreditasyon Derneği (The Association for Evaluation and Accreditation of Medical Education Programs)
CHERRIES: Checklist for Reporting Results of Internet E-Surveys
